# Synthetic peptide matrices as support for stem cells culture

**DOI:** 10.1186/1753-6561-9-S9-P50

**Published:** 2015-12-14

**Authors:** Youlia Serikova, Martin Bousmanne, Jean-Christophe Drugmand, Marc Fouassier, Laurent Jeannin, Yves-Jacques Schneider

**Affiliations:** 1 Institute of Life Sciences, Université catholique de Louvain, Louvain-la-Neuve, 1348, Belgium; 2 Peptisyntha SA, Brussels, 1120, Belgium; 3 Pall Life Sciences, Brussels, 1120, Belgium

## Background

Nowadays, stem cells draw great interest in regenerative medicine[1].However, due to low occurrence in tissues, their in vitro expansion is required to gain access to therapeutic applications [2].

Furthermore, the existing culture systems often include xeno-derived components, present upscaling limitations [3, 4] and do not necessary address important considerations, such as those related to biosafety, availability and homogeneity of (biological) raw material and process reproducibility. Moreover, the regulatory guidelines focus on the importance of defined environments, minimal handling and continuity between development and industrialization scales[5, 6]. In this context, new scalable solutions a reactively considered in order to achieve production of stem cells at higher densities[7].Among these, synthetic peptides are of particular interest[4] as potential alternatives to xeno-derived culture supports, able to mimic the physico-biochemical properties of natural cell matrices.

In this way our work aims at assessing new xeno-free culture matrices based on short, synthetic and bioactive peptides and demonstrating the scalability, reproducibility and performance of such peptides-based matrices for human stem cells culture. This report is focused on a part of the work performed to study the stability of synthetic peptide coatings on polymer surfaces and the capability of such coatings to favour stem cells adhesion and growth.

## Materials and methods

Experimental peptides, i.e. short bioactive sequences derived from RGD (P1), collagen I (P2), fibronectin (P3) orlaminin (P4), were designed and synthesized at Peptisyntha.

Peptide coatings were prepared from P1, P2, P3 and P4 on culture polystyrene (PS). Coated supports were washed, dried and physico-chemical characterization of these coatings was conducted using X-Ray Photoelectron Spectroscopy (XPS), contact angles measurements and Time of Flight-Secondary Ions Mass Spectrometry (ToF-SIMS). Then, the stability of coatings was analysed using High Performance Liquid Chromatography (HPLC) by determining the remaining peptides in solution after coating. The topography of coated supports was also analysed by Scanning Electron Microscopy (SEM). This analysis was performed on PS samples that were coated, dried and carbon sputtered.

The efficiency of peptide coatings was subsequently analysed using human Adipose tissue-Derived Stem Cells (hADSCs). hADSCs were seeded at 5 000 cells/cm2 in serum-free medium (Basal Defined Medium)and incubated until 24h. Medium was then supplemented with serum and cells were cultured till 5 days. Cell adhesion efficiency was followed using phase contrast microscopic observations. Cells were also fixed with 4% (v/v) formaldehyde stained with DAPI (nuclei) and rhodaminephalloidin (actin filaments) and cell density was determined by fluorescence microscopy (NIS software analysis).

## Results

Firstly, the physico-chemical characterization enabled to identify the peptides presence at PS surfaces after coating. Indeed, the surface chemical composition was analysed by XPS (Table 1). Oxygen to carbon and nitrogen to carbon atomic percentage ratios on coated versus uncoated PS were determined. Peptide coatings induced spectra shift and atomic percentage ratios increase, due to the peptides presence. In addition, ToF-SIMS analyses evidenced the presence of characteristic amino acids derived from the peptide sequences on coated surfaces. These results evidenced the peptides persistence on coated PS surface.

**Table 1:** Summary of obtained results for uncoated (PS),wetted (PBS) or coated surfaces by XPS analysis of surface chemical composition^1^, contact angles measurements of surface wettability^2^, HPLC dosages of remaining peptides in solution after coating^3^, hADSCs adhesion efficiencies assessment(6h after seeding)^4^and hADSCsPDL determination after 5 days of culture^5^.

	*XPS^1^* *O/C* *(N = 3, n = 3)*		*Contact angles (°)^2^* *(N = 3, n = 15)*	*HPLC (% of remaining peptides)^3^* *(N = 2, n = 3)*	*Adhesion* *(% ofadherent cells)^4^* *(N = 3, n = 3)*	*Growth (PDL)^5^* *(N = 3, n = 3)*
***PS (control)***	(**124** ± 12) × **10^-3^**	(**7** ± 3) × **10^-3^**	**81.5** ± 0.6	/	/	/
***PBS (control)***	/	/	**60.3** ± 2.3	/	**49.7** ± 2.0	**1.76** ± 0.08
***BSA***	/	/	**51.4** ± 3.2 *	/	**9.6** ± 1.0*	< 0*
***Collagen I***	/	/	/	/	**63.1** ± 1.6*	**2.12** ± 0.04
***Fibronectin***	/	/	/	/	**44.1** ± 1.1	**1.90** ± 0.04
***Puramatrix®***	/	/	/	/	**47.9** ± 2.3	**1.66** ± 0.09
***P1***	(**174** ± 18) × **10^-3^**	(**44** ± 3) × 10^-3^*	**35.5** ± 0.8*	**71.6** ± 5.5	**61.6** ± 1.9*	**2.13** ± 0.06
***P2***	(**204** ± 13) × **10^-3^**	(**27** ± 5) × **10^-3^**	**25.8** ± 1.5*	**87.9** ± 2.3	**66.7** ± 2.1*	**1.80** ± 0.03
***P3***	(**282** ± 47) × 10^-3^*	(**63** ± 10) × 10^-3^*	**19.6** ± 0.7*	**63.8** ± 4.8	**69.2** ± 2.3*	**1.90** ± 0.08
***P4***	(**141** ± 13) × **10^-3^**	(**55** ± 13) × 10^-3^*	**14.8** ± 0.8*	**51.2** ± 4.7	**54.8** ± 2.0	**2.07** ± 0.08

Results are means ± standard error of the mean. (Values were statistically compared to control conditions, α=0.05).

Additionally, the peptide coatings stability was studied by HPLC analysis of remaining peptides in solutions after PS coating (Table 1). Coating, post-coating, washing and 5 days washout solutions were analysed. The peptide concentrations decreased from initial coating to post-coating solution. This decrease varied depending on the peptide sequence. It means that a part of introduced peptides remained at the polymer surface after coating and this immobilization was stable in time since peptide amounts in washing and washout solutions were negligible.

Furthermore, surfaces hydrophilicity was assessed by static contact angles measurements (Table 1) on control, pre-wetted (in Phosphate Buffered Saline - PBS), and coated PS surfaces. Additionally, Bovine Serum Albumin (BSA) coating was performed to assess "passivation" effect. It was observed that pre-wetting induced a slight decrease in contact angles, suggesting that modifications occur at hydrophilised PS surface in contact with saline buffer. BSA-coated surfaces presented lower contact angles than for pre-wetted surfaces, while coatings increased the surfaces hydrophilicity (contact angles decrease) in comparison with all the other conditions. Increased hydrophilicity of coated surfaces could favour interactions between culture supports and cells.

Besides these analyses, coatings topography was studied by SEM. Raised area and networks were observed on coated PS surfaces but not at the control ones. Variations in topography from one coating to another were observed and it could be related to different sequences, which confers different behaviours to the peptides.

Afterwards, peptide coatings efficiencies vs. control supports or matrices were studied using hADSCs. hADSCs adhesion was analysed after 6h of culture (Table 1). Indeed, control surface was compared to animal-derived (BSA, collagen, fibronectin) or synthetic coatings (Puramatrix® or experimental peptides at same concentrations). It was observed that peptide coatings improved cell adhesion in serum-free conditions, in comparison to control matrices. Moreover, population-doubling levels (PDL) were calculated after 5 days of culture (Table 1) in a serum-supplemented medium. Peptide coatings also promoted cell growth but their effect was variable, depending on bioactive sequence and less pronounced than for adhesion (possibly due to serum presence).

**Figure 1:**
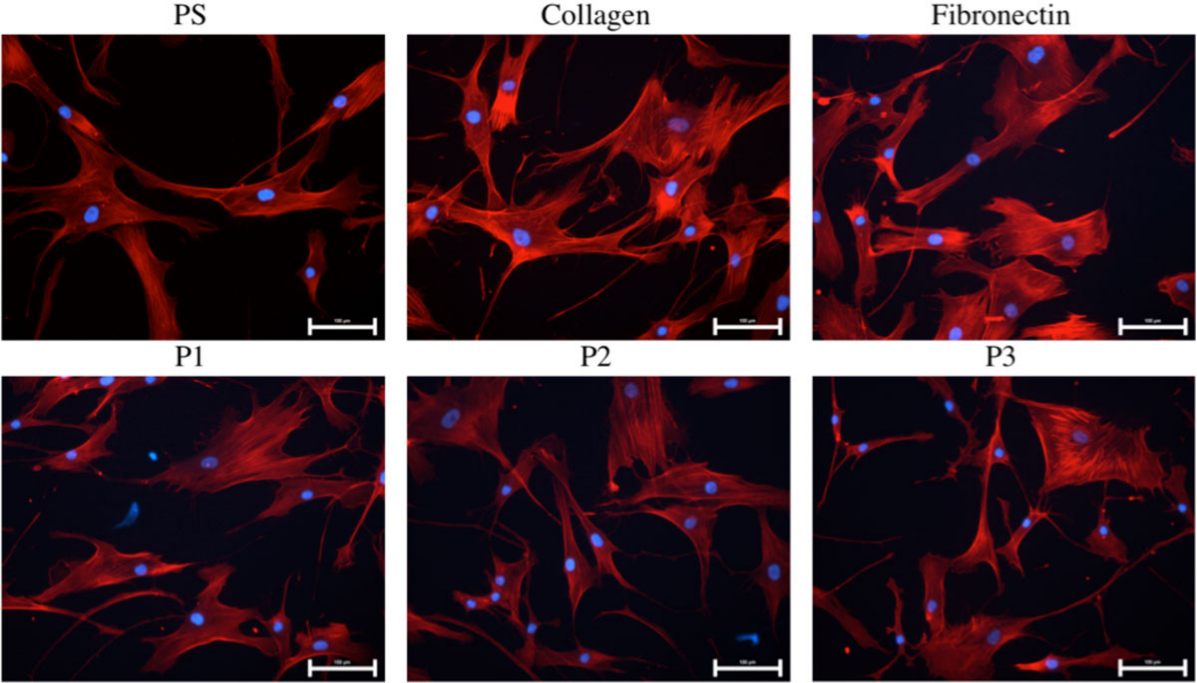
**Fluorescence microscopy pictures of hADSCs after 24 hours of culture on several supports (bar= 100 μm)**. Staining: DAPI (blue - nuclei) and rhodaminephalloidin (red - actin filaments).

Finally, hADSCs cultured on peptide coatings vs. control surfaces were compared by fluorescence microscopy. This analysis enabled to observe that peptide coatings were compatible with these cells and induced similar hADSC's morphology (Figure 1), phenotype and spreading in comparison with control surfaces.

## Conclusion

This work demonstrates the ability to stably coat cell culture supports with cytocompatible and xeno-free matrices able to promote stem cells adhesion and growth.

Importantly, peptide coatings increase the wett ability of culture supports and the peptide sequences seem to differently influence the behaviour at the coated surfaces, which suggests that each experimental peptide could have higher specificity towards certain cell types.

As a conclusion, besides the capability of producing the experimental peptides at large scale and within affordable cost, the latest results bring additional conviction that the studied peptides constitute interesting paths to the development of xeno-free matrices in order to achieve large-scale stem cells production.

## Acknowledgements

This work was funded by Innoviris (Brussels Region).
